# Merging Experimental Design and Nanotechnology for the Development of Optimized Simvastatin Spanlastics: A Promising Combined Strategy for Augmenting the Suppression of Various Human Cancer Cells

**DOI:** 10.3390/pharmaceutics14051024

**Published:** 2022-05-09

**Authors:** Shaimaa M. Badr-Eldin, Hibah M. Aldawsari, Nabil A. Alhakamy, Usama A. Fahmy, Osama A. A. Ahmed, Thikryat Neamatallah, Singkome Tima, Raghad H. Almaghrabi, Fayda M. Alkudsi, Asmaa A. Alamoudi, Amjad A. Alzahrani, Sabna Kotta, Omar D. Al-hejaili

**Affiliations:** 1 Department of Pharmaceutics, Faculty of Pharmacy, King Abdulaziz University, Jeddah 21589, Saudi Arabia; haldosari@kau.edu.sa (H.M.A.); nalhakamy@kau.edu.sa (N.A.A.); uahmedkauedu.sa@kau.edu.sa (U.A.F.); oaahmed@kau.edu.sa (O.A.A.A.); ralnemari0010@stu.kau.edu.sa (R.H.A.); fkudsi@stu.kau.edu.sa (F.M.A.); aalamoudi0285@stu.kau.edu.sa (A.A.A.); aalzhraani0685@stu.kau.edu.sa (A.A.A.); skotta@kau.edu.sa (S.K.); omer.d.alhejaili@gmail.com (O.D.A.-h.); 2 Center of Excellence for Drug Research and Pharmaceutical Industries, King Abdulaziz University, Jeddah 21589, Saudi Arabia; 3 Mohamed Saeed Tamer Chair for Pharmaceutical Industries, King Abdulaziz University, Jeddah 21589, Saudi Arabia; 4 Department of Pharmacology and Toxicology, Faculty of Pharmacy, King Abdulaziz University, Jeddah 21589, Saudi Arabia; taneamatallah@kau.edu.sa; 5 Department of Medical Technology, Faculty of Associated Medical Sciences, Chiang Mai University, Chiang Mai 50200, Thailand; singkome.tima@cmu.ac.th

**Keywords:** combined mixture-process variable design, spanlastics, simvastatin, optimization, in vitro cytotoxicity

## Abstract

Simvastatin (SMV) is an antihyperlipidemic agent that has been investigated as a possible anti-cancer agent. An obstacle to malignant tumor therapy using drugs is the delivery of adequate levels to the cancer cells while minimizing side effects following their systemic administration. To circumvent this challenge, the researchers directed towards the field of nanotechnology to benefit from the nano-size of the formulation in passively targeting the tumor cells. Thus, our study aimed at investigating the potential of a combined mixture–process variable design for optimization of SMV spanlastics (SMV-SPNs) with minimized particle size and maximized zeta potential to enhance the anticancer activity of the drug. The study investigated the effects of Span^®^ 20 and Tween^®^ 80 as mixture components and sonication time as a process variable on particle size, polydispersity index, and zeta potential as responses. SPNs were prepared using an ethanol injection method. Combining the predicted optimized variables’ levels is supposed to achieve the set goals with a desirability of 0.821. The optimized spanlastics exhibited a measured globule size of 128.50 nm, PDI of 0.329, and ZP of −29.11 mV. The percentage relative error between predicted responses and the observed ones were less than 5% for the three responses, indicating the optimization technique credibility. A significant improvement in the cytotoxicity of the optimized formulation against three different cancerous cell lines was observed in comparison with SMV. The inhibitory concentration (IC_50_) values of MCF-7, HCT-116, and HEPG2 were found to be 0.89, 0.39, and 0.06 μM at 24 h incubation. The enhanced cytotoxicity could be assigned to the possible improved permeation and preferential build-up within the cancerous cells by virtue of the minimized size. These findings imply that SMV-SPNs could be an ideal strategy to combat cancer.

## 1. Introduction

Cancer is a heterogeneous illness that could rapidly progress to an unmanageable stage after it first develops [1]. It is one of the major causes of mortality around the world, with millions of new cases recorded each year [2]. Chemotherapeutic drugs are the most popular approach to treating cancer patients because of their ability to limit the uncontrolled development of malignant cells [3]. The main drawbacks of chemotherapeutic agents are non-specificity and the development of multidrug resistance during therapy [4]. Accordingly, there are numerous undesirable side effects, as well as insufficient drug delivery in most cases [5].

Simvastatin (SMV) is widely used for the treatment of patients suffering from dyslipidaemia via the inhibition of the HMG-COA reductase enzyme. Owing to its poor water solubility, reduced intestinal uptake, and exposure to extensive presystemic metabolism, SMV suffers from poor oral bioavailability [6]. Thus, researchers directed towards investigating alternative routes for the drug administration, including the transdermal one to surpass such pitfalls [7].

Recently, statins have been identified as possible anti-tumour agents against several types of cancer cells [8,9]. However, an obstacle to malignant tumour therapy lies in the challenge of delivering the appropriate concentrations of drugs to the cancer cells while minimising non-specific toxicity incidence resulting from minimal selectivity following administration, in addition to the liability of developing drug-resistance by the cancer cells [10]. This could be overcome by applying nanotechnology to passively target drugs to tumour cells. Nano-sized drug delivery systems can readily penetrate cancerous growths, with subsequent accumulation resulting from poor lymphatic drainage of the tumors. Thus, nano-sized systems are considered a strategy of interest for cancer therapy [11]. Besides adequate specificity, cancer nanotechnology provides additional advantages of high drug entrapment as well as high tolerability compared with conventional chemotherapeutic agents [12]. Additional advantages of nano-sized delivery systems include a large surface area that leads to improved drug dissolution, proper cellular uptake because of their small size, a long circulation time in blood, and physical stability [13]. In vitro cytotoxicity studies on cancer cell lines represent a potential strategy for screening the anticancer activity of such formulations against various types of cancer.

Spanlastics (SPNs) are surfactant-based nanovesicles with an amphiphilic nature that allows them to trap the drug in the bilayer’s core cavity. They are chemically stable, and they possess elasticity and deformability characteristics because of the incorporation of an edge activator. In addition, they possess the advantages of being biodegradable, non-immunogenic, target-specific, and able to enhance the bioavailability and stability of entrapped drugs [14].

Traditional experiments consume time and effort in the development of complex formulations. Accordingly, the use of a statistical design and modelling approach is recommended in such cases. The optimization of formulations often need to assess both the mixture components of the formulation and the process variables affecting the responses synchronously. A combined mixture–process variable design is beneficial in such a case [15]. To this end, the potential of a combined mixture–process variable design (CMPV) for the prediction of the optimized SMV-SPNs with minimized particle size and maximized zeta potential was explored. Cytotoxicity studies demonstrated that the optimized SMV-SNPs significantly reduced the viability of MCF-7, HCT-116, and HepG2 cancer cells in comparison with SMV as confirmed by the significantly low IC_50_ values.

## 2. Materials and Methods

### 2.1. Materials

Simvastatin was purchased from Qingdao Sigma Chemical Co., Ltd. (Qingdao, China). Span^®^ 20 and Tween^®^ 80 were purchased from Sigma-Aldrich (GmbH, Germany). All other chemicals and solvents were of analytical grade.

### 2.2. Combined Mixture-Process Variable Design

Combined two-component mixture, one process variable design (CMPV) was utilized for the formulation and optimization of SMV-SPNs. This approach allows for assessing how the responses are synchronously influenced by both the mixture components (MCs) of the formulation and the process variable (PV). In this study, the two components of the SPNs were Span^®^ 20 (X_1_) and Tween^®^ 80 (X_2_). Both components were used in the range of 1–9 parts so that the total mixture is 10 parts. Sonication time (Z_1_) was studied as process variable (PV) in the range of 0–10 min. All other process variables including stirring speed, time, and temperature were kept constant. Particle size (PS, nm) (Y_1_), polydispersity index (PDI) (Y_2_), and Zeta potential (ZP, mV) (Y_3_) were the measured response variables. The MCs and PV with their corresponding ranges, in addition to the response variables and the constraints set in the optimization process are presented in Table 1. Design Expert^®^ software (Version 11.0, Stat-Ease Inc., Minneapolis, MN, USA) was employed for generating the design points and statistically analyzing the responses. The design points were chosen on the basis of the D-optimal design where the total number of design points was 17 including 3 replicate points and additional center point in addition to the required model and lack of fit points. Analysis of variance was employed to assess the impact of the MCs and PV as well as their interaction on the responses at 95% level of significance. One factor and three-dimensional response plots were constructed to display such effects and interactions.

### 2.3. Preparation of SMV-SPNs

SPNs were prepared using ethanol injection method [16,17]. First, the drug (20 mg) and Span were dissolved in 5 mL absolute ethanol. Then, the alcoholic solution was rapidly injected into 10 mL aqueous solution of edge activator (Tween 80) prepared at a temperature of 70 °C. The amounts of Span and Tween 80 were calculated as per the experimental design. The solution was kept on a magnetic stirrer revolving at 1000 rpm at the same temperature for 30 min to allow for solvent evaporation. The formed dispersion was ultra-sonicated for the specified time according to the design after volume adjustment to 10 mL.

### 2.4. Optimization of SMV-SPNs

To anticipate the optimized levels of the mixture components as well as the process variable, numerical optimization and desirability function were utilized. The goal of the optimization process was to obtain the smallest possible SPNs size and PDI, in addition to the highest absolute ZP value.

### 2.5. Characterization of SMV-SPNs

#### 2.5.1. PS, PDI, and ZP Measurement

PS (z-average), PDI, and ZP of SMV-SPNs were measured for all the prepared formulations using Zetasizer Nano ZSP (Malvern Panalytical Ltd., Malvern, UK) after appropriate dilution. Each measurement was presented as the mean of five runs.

#### 2.5.2. Transmission Electron Microscope (TEM)

The optimized SMV-SPNs were visualized using JEOL GEM-1010 (JEOL Ltd., Akishima, Tokyo, Japan) transmission electron microscope (TEM) at 80 kV at The Regional Center for Mycology and Biotechnology (RCMB) Al-Azhar University, Cairo, Egypt. One drop of diluted SPNs sample was put on a carbon-coated grid, which was then allowed to dry at temperature of 25 ± 0.5 °C. Further, the sample was negatively stained with 1% phosphotungstic acid and then dried for 20 min at room temperature before being visualized.

### 2.6. In Vitro Cytotoxicity of Optimized SMV-SPNs

#### 2.6.1. Cell Culture

Human breast cancer cell line (MCF-7), colorectal cell line (HCT-116), and liver cancer cell line (HepG2) were obtained from American Type Culture Collection (ATCC, Rockville, MD, USA). The cells were cultured in Dulbecco’s Modified Eagles Medium (DMEM) supplemented with 10% (*v/v*) fetal bovine serum (FBS), 10,000 units/mL penicillin/streptomycin, and 1% (*v/v*) L-glutamine at 37 °C in humidified 5% CO_2_ incubator. 

#### 2.6.2. Cytotoxicity Assay

The cytotoxicity was assessed using the MTT assay as previously described [18]. Cells were seeded in 96-well plates at a density of (5 × 10^3^ cells/well) and left to attach overnight. Subsequently, treatment of the cells with SMV, SMV-SPNs, and blank SPNs for 24 h at concentration range (0.01–100 µM) was performed. Treatments were removed and 100 µL of MTT solution (2 mg/mL) was added to each well and incubated the cells at 37 °C for 4 h. The formazan crystals formed were dissolved in DMSO (100 µL) and absorbance was measured at 570 nm on a plate reader (Tecan Group Ltd., Seestrasse, Maennedorf, Switzerland). The results were expressed as the percentage of viable cells in relation to the untreated cells (control). The data were obtained from three independent experiments (*n* = 3).

## 3. Results and Discussion

### 3.1. Model Fit Statistical Analysis

Table 2 summarizes the combination of variables in each experimental run and the corresponding responses. Fit statistics analysis was performed for each response individually to obtain a CMPV polynomial model describing the relation between this response and the studied MCs and PV. The software suggests the best fitting model for every response based on maximizing the Adjusted R^2^ and the lowest predicted residual error sum of squares (PRESS). According to the model fit statistics, presented in Table 3, the suggested model was Quadratic × Linear (Q × L) for the three responses. The predicted R^2^ reasonably coincides with the adjusted R^2^ (the difference is less than 0.2) for all responses indicating the model suitability. In addition, an adequate precision of more than four indicates an appropriate signal to noise ratio. Accordingly, the Q × L model is proven to be appropriate for the exploration of the experimental design space.

### 3.2. Diagnostics Analysis

**Figure 1:**
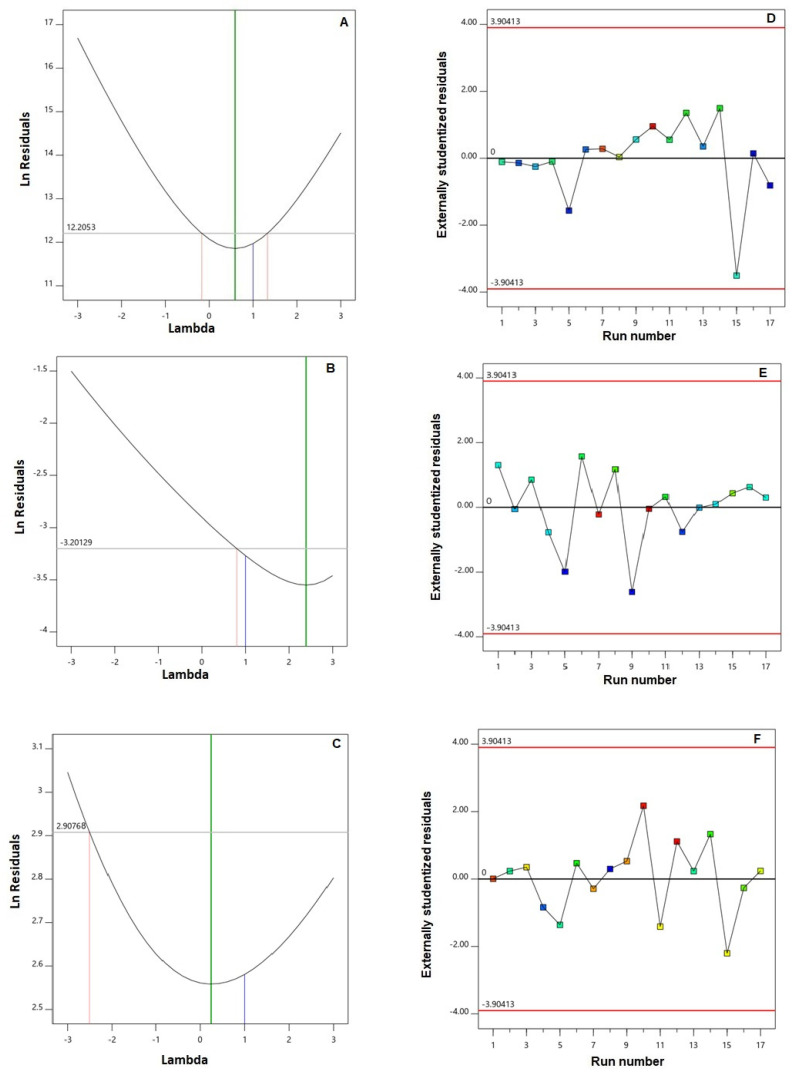
Diagnostic plots for the quadratic × linear model of the particle size (**A**,**D**), polydispersity index (**B**,**E**), and zeta potential (**C**,**F**) of SMV-SPNs (Box–Cox plot for power transforms (**A**–**C**); externally studentized residuals vs. run number plot and (**D**–**F**)). **Abbreviations:** SMV, simvastatin; SPNs, spanlastics.

For establishing the goodness of fit for the investigated responses to the Q × L model, diagnostic plots were created. Figure 1A–C, representing the Box–Cox plot for power transforms, demonstrates a best lambda (λ) value of 0.59, 2.39, and 0.25 (shown by the green line) for Y_1_, Y_2_, and Y_3_, respectively. The computed confidence interval (represented by the red lines) comprises the value one (current λ for all responses represented by the blue line); accordingly, no specific data transformation is required [19]. The lack of requirement for transformation is corroborated by the maximum to minimum measured responses, where a ratio greater than 10 shows that transformation is required. Furthermore, the residual vs. run plots, shown in Figure 1D–F show randomly scattered points, indicating that no hidden variable exists and could exert an influence on any of the measured responses [20,21].

### 3.3. Polynomial Equations for the Investigated Responses

The polynomial equations representing the responses in terms of L-Pseudo components of the mixture and coded factor for the process variable were generated as follows:

Y_1_ (PS) = 588.80 X_1_ + 402.16 X_2_ − 1326.05 X_1_X_2_ − 216.51 X_1_Z_1_ − 242.41 X_2_Z_1_ + 550.07 X_1_X_2_Z_1_

Y_2_ (PDI) = 0.4481 X_1_ + 0.3519 X_2_ − 0.3492 X_1_X_2_ − 0.1741 X_1_Z_1_ − 0.0594 X_2_Z_1_ + 0.4741 X_1_X_2_Z_1_

Y_3_ (ZP) = 30.37 X_1_ + 21.41 X_2_ + 7.84 X_1_X_2_ + 0.422 X_1_Z_1_ + 2.19 X_2_Z_1_ − 8.24 X_1_X_2_Z_1_

The coded equations are beneficial for pointing out the relative influence of the factors by the comparison of their coefficients. The first three terms of each equation containing the MCs only (X_1_ and X_2_) represent the mixture properties at the mid-value of the PV (sonication time of 5 min that is the coded level set at zero). The last three terms shows the linear effect of the studied PV (Z_1_) on the mixing properties of the MCs that shifts the mean response at any given combination of MCs with the variable Z_1_ variation from the coded level 0 to +1 [15,22]. The presence of significant MPV coefficients in the equations highlights the usefulness of employing the CMPV design as it reveals the interaction between the MCs and the PV; such an interaction could never be detected using the traditional one factor at a time approach or even experimental designs done individually on MCs and PVs [22,23].

### 3.4. Influence of Variables on PS (Y_1_) and PDI (Y_2_)

**Figure 2:**
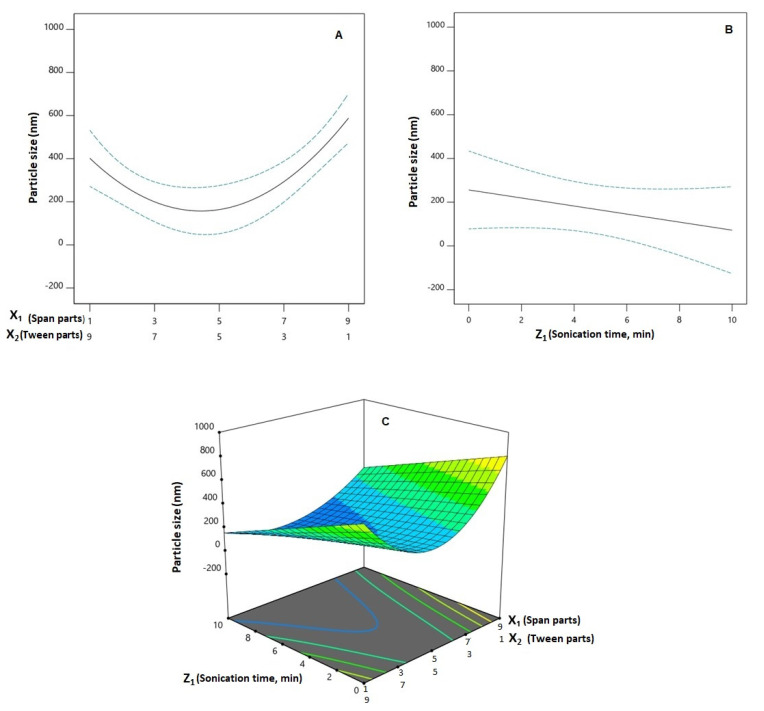
One-factor plots for the effect of the binary mixture components (**A**) and the sonication time (**B**) at the mid-values of the other variables on particle size (Y_1_); three-dimensional mixture-process plot (**C**) for the interaction between mixture components and sonication time. **Abbreviations**: SMV, simvastatin; SPNs, spanlastics; X_1_, Span 20 parts; X_2_, Tween 60 parts (X_1_ and X_2_ add up to 10 parts).

**Figure 3:**
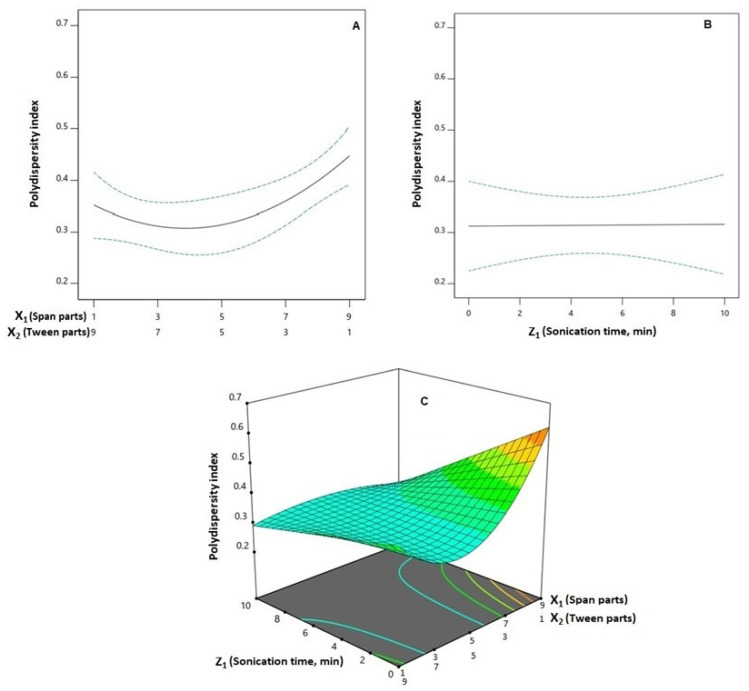
One-factor plots for the effect of the binary mixture components (**A**) and the sonication time (**B**) at the mid-values of the other variables on polydispersity index (Y_2_); three-dimensional mixture–process plot (**C**) for the interaction between mixture components and sonication time. **Abbreviations**: SMV, simvastatin; SPNs, spanlastics; X_1_, Span 20 parts; X_2_, Tween 60 parts (X_1_ and X_2_ add up to 10 parts).

Preferential dissemination within malignant tissues has been reported for nanoparticulate systems with sizes smaller than 400 nm [24,25]. However, inefficient tumor invasion, possibly caused by pathological features produced by the cancerous growth, may offset the preferred accumulation of the nano-particulate systems and their concomitant therapeutic outcome [26]. In addition, PDI, as a measurement of particle size distribution, indicates dispersion homogeneity. It is reported that a highly monodisperse system exhibits a PDI less than 0.05, while a PDI greater than 0.7 indicates a heterogeneously distributed system [27]. Thus, preparing SPNs with the lowest particle size and PDI was one of the goals of this study. For the prepared SPNs, the mean PS showed a wide variation ranging from 74.45 ± 3.16 to 891.80 ± 36.89 nm as shown in Table 2, while the PDI ranged from 0.216 ± 0.018 to 0.620 ± 0.057, indicating an acceptable size distribution. Analysis of variances (ANOVA) revealed the significance of the Q × L model for both responses (*p* = 0.0005 and 0.0006, respectively). The computed *F*-values of 10.78 and 11.64 for particle size and PDI, respectively, indicate the significance of the model; there is only a likelihood of 0.05% and 0.06% that these *F*-values could be this large owing to noise. The lack of fit *F*-values of 0.9612 and 0.1893 for both responses show a non-significant lack of fit in relation to the pure error, indicating fitting of the data to the model. According to the computed *p*-values, the linear mixture terms; X_1_ and X_2_ were significant on both sizes (*p* = 0.0054) and PDI (*p* = 0.0084). In addition, the interaction terms X_1_X_2_ (*p* = 0.0006 for Y_1_ and *p* = 0.0286 for Y_2_), X_1_Z_1_ (*p* = 0.0038 for Y_1_ and *p* = 0.0001 for Y_2_), and X_2_Z_1_ (*p* = 0.0051 for Y_1_ and *p* = 0.0109 for Y_2_) were significant on both responses. Furthermore, the term X_1_X_2_Z_1_ was significant on the PDI (*p* = 0.0180). The effect of the binary mixture components and the sonication time at the mid-values of the other factor, in addition to the three-dimensional mixture–process plot for the PS and PDI are graphically illustrated in Figure 2 and Figure 3, respectively.

It was evident that the PS decreases with increasing Span proportion at its lower levels; on the other hand, the size shows a significant increase with increasing Span proportion at the higher levels. A similar corresponding behavior was observed with Tween being the second component of the mixture. This observation coincides with previous studies that reported the decrease in PS with increasing edge activator percentage; the researchers attributed this decrease to reduced interfacial tension that facilitates particles partition to yield smaller particles [28,29].

It is worthy to note that the formulations generally prepared at higher levels of Span generally showed higher PS compared to those with higher levels of Tween at the same sonication time. This could be attributed to the Span hydrophobic side chain. A steric repulsion occurs at higher levels of Span that leads to increase the formed SPNs size. On the other hand, higher tween levels facilitate assembly of the SPNs with lower steric repulsion compared to the same levels of Span. This requires further and detailed investigation to understand this behavior and prove this postulation. The different trend observed at higher Span proportions highlights the marked role of the interaction between the MCs and the studied PV. Increasing sonication time is previously reported to reduce the particle size of the vesicular systems [30,31,32]. The effect of sonication could be attributed to the cavitation (compression) forces generated by the ultrasonic waves passage through the vesicular dispersion leading to the fractionation of the particles with a consequent reduction in their sizes [33].

### 3.5. Influence of Variables on Zeta Potential (ZP, Y_3_)

**Figure 4:**
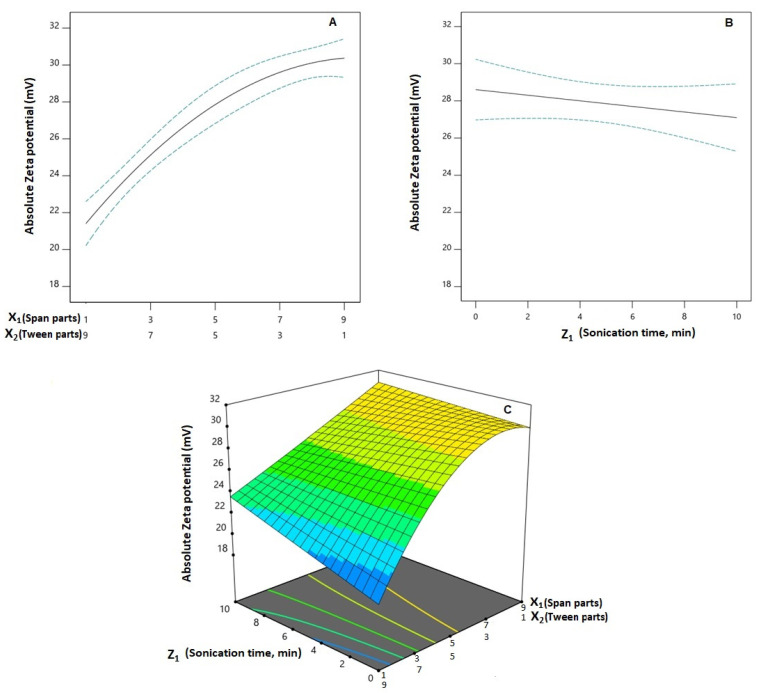
One-factor plots for the effect of the binary mixture components (**A**) and the sonication time (**B**) at the mid-values of the other variables on absolute zeta potential (Y_3_); three-dimensional mixture-process plot (**C**) for the interaction between mixture components and sonication time. **Abbreviations**: SMV, simvastatin; SPNs, spanlastics; X_1_, Span 20 parts; X_2_, Tween 60 parts (X_1_ and X_2_ add up to 10 parts).

Increased absolute zeta potential values are expected to impart physical stability to the dispersed delivery systems and minimize aggregation owing to increased electrostatic repulsion [34]. The prepared spanlastics possess a negative zeta potential, which ranged from −19.40 ± 1.34 to −31.70 ± 2.49 mV, and could originate from the partially negative groups available in the polar head of Span. These polar heads are normally directed to the external aqueous phase, imparting a net negative ZP for the prepared vesicles [29]. As per the ANOVA analysis, the Q × L model was significant for the ZP absolute values (*p* < 0.0001). The computed *F*-values of 33.46 indicate the significance of the model; there is only a likelihood of 0.01% that the *F*-value could be this large in credit to noise. Lack of fit *F*-values of 2.06 show a non-significant lack of fit in relation to the pure error indicating fitting of the data to the model. According to the computed *p*-values, the linear mixture terms X_1_ and X_2_ were significant on ZP (*p* < 0.0001). The interaction term X_1_X_2_ is related to the interaction between MCs, in addition to the interaction terms X_2_Z_1_, and X_1_X_2_Z_1_ being related to the interactions between the MCs and the PV that were significant on the ZP (*p* = 0.0103, 0.0055, and 0.0252, respectively). The effect of the binary mixture components and the sonication time at the mid-values of the other factors, in addition to the three-dimensional mixture–process plot for ZP, are graphically illustrated in Figure 4. It was evident that the absolute value of ZP increases with an increasing Span proportion.

### 3.6. Optimization Using Numerical Approach

**Figure 5:**
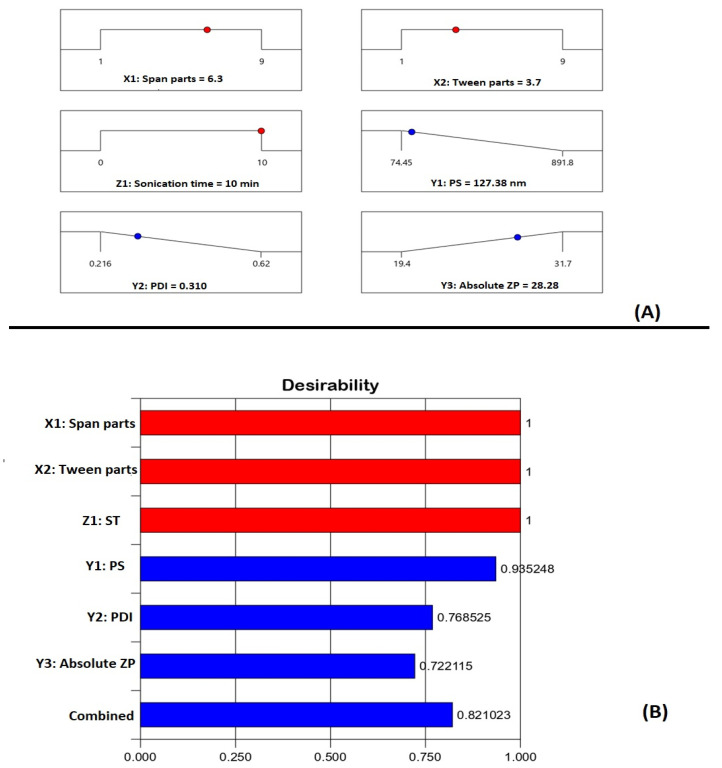
(**A**) Ramp graphs representing the optimized levels of Span 60, Tween 80, and sonication time, in addition to the predicted responses for the optimized SMV-SPNs. (**B**) Desirability values for the predicted responses and overall desirability of the optimized SMV-SPNs. **Abbreviations:** SMV, simvastatin; SPNs; spanlastics; ST, sonication time (min); PS, particle size; PDI, Polydispersity index; ZP, zeta potential.

The goal of pharmaceutical formulation optimization is to forecast the levels of variables that will result in a product with the desired qualities. The optimization process in this study aims at decreasing particle size and PDI to the lowest possible value with simultaneous maximizing of the ZP absolute value of the proposed SMV-SPNs. The numerical optimization technique was adopted to anticipate the levels of the MCs and the PV that upon combination could achieve the previously set goals with the highest possible desirability. The ramp graphs presented in Figure 5A shows the optimized levels and the predicted responses, while the desirability for each response and the overall desirability are graphically illustrated in Figure 5B. The measured responses were 128.50 nm, 0.329 for PDI, and −29.11 for ZP. The percentage relative error between predicted responses and the observed ones were less than 5% for the three responses (0.87, 4.44, and 2.93 for PS, PDI, and ZP, respectively). This relatively low error percentage proves the optimization technique credibility.

### 3.7. Transmission Electron Microscopy (TEM)

**Figure 6:**
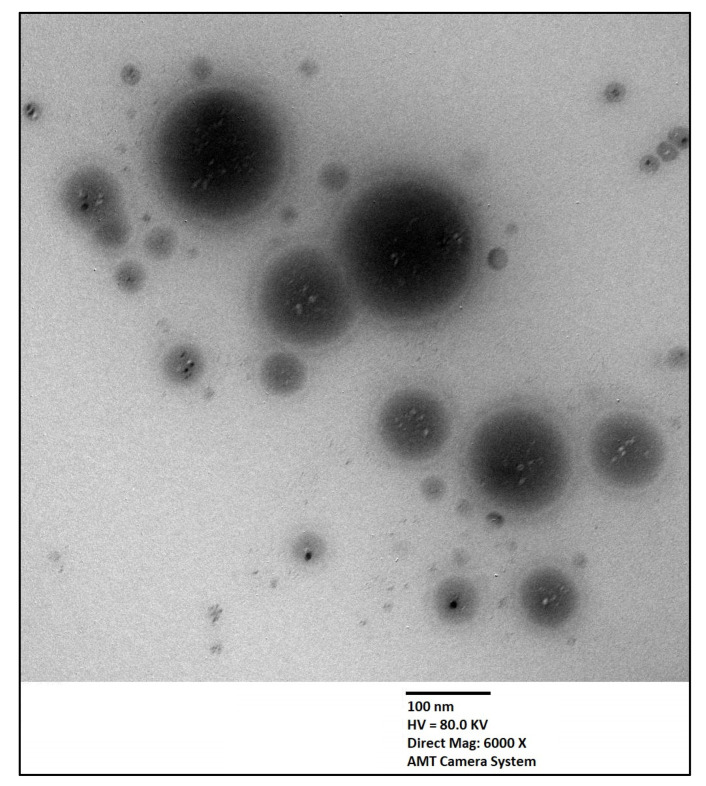
Transmission electron microscope micrograph of the optimized SMV-SPNs. **Abbreviations:** SMV, simvastatin; SPNs; spanlastics.

The shape of the optimized SMV-SPNs was visualized using TEM as depicted in Figure 6. The TEM micrographs show spherical vesicles with rounded contours. The size of the vesicles well coincide with that measured by the dynamic light scattering technique. El-nabarawy et al. [35] reported a similar spherical shape for zolmitrptan spanlastic vesicles.

### 3.8. In Vitro Cytotoxicity

**Figure 7:**
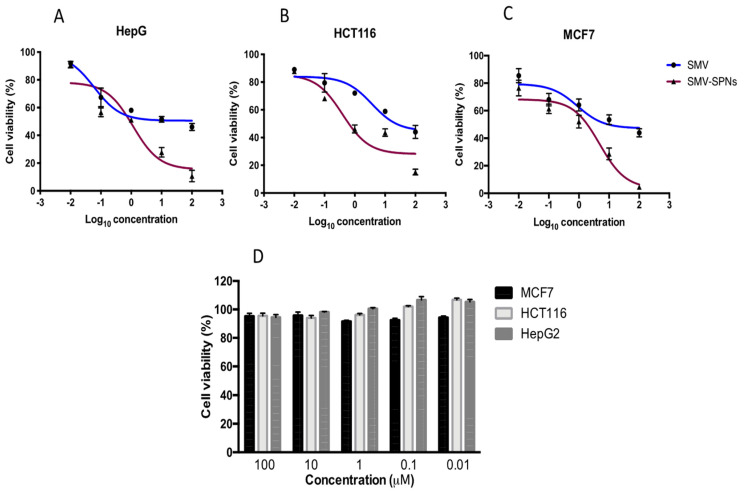
Cell viability evaluation using the MTT assay after 24 h of treatment with SMVor SMV-SPNs (**A**) MCF-7 (**B**) HCT-116 (**C**) HEPG2 cells (**D**) cell viablitiy after 24 h of treatment with blank-SPNs. Data are expressed as the mean ± SEM (*n* = 3). **Abbreviations:** SMV, simvastatin; SPNs; spanlastics.

The antiproliferative effect of the SMV and SMV-SPNs on the viability of MCF-7, HCT-116, and HepG2 cells was examined using MTT assays. As displayed in Figure 7D, more than 90% of the cells were viable after exposure to blank SNPs suggesting a non-significant reduction in the cell viability. SMV treatment (0.01–100 µM) significantly reduced cell viability in a concentration-dependent manner (*p* < 0.05). Several mechanisms of action have been proposed for simvastatin-induced cytotoxicity, mainly the direct suppression of cholesterol synthesis particularly by inhibiting HMG-CoA Reductase and isoprenylation as well as the inhibition of Ras, an activated protein in several cancers [36,37]. SMV-SPNs further reduced the viability of the cells showing a significant cytotoxic effect in comparison to SMV (*p* < 0.05) (Figure 7A–C). The calculated IC_50_ for SMV and SMV-SPNs are presented in Table 4. This potential effect of SMV-SPNs on cancer cells can be assigned to the possible enhanced cellular uptake and preferential build-up within the cancerous cells by virtue of the minimized size and role of the edge activator (surfactant) present in the formulation. The edge activator could potentially improve the drug permeability via biological membranes; in addition, it could increase the vesicles bilayer fluidity; thus, enabling their facile diffusion through the cellular membrane with consequent drug build-up inside the cells [38]. Our finding of spanlastic vesicle ability to enhance the efficacy of SMV coincides with previous research. For example, Sodium valproate nanospanlastics have been developed by Badria et al. [39] as a successful platform for treating alopecia. Alhakamy et al. reported the enhanced antifungal activity of luliconazole via the development of an optimized spanlastics formulation. Furthermore, Alaaeldin et al. [40] reported enhanced anticancer activity of thymoquinone spanlastics against MCF-7 cells as compared to either free drug or conventional liposomes that were attributed similarly to augmented cellular uptake and permeation. Considering the proposed molecular mechanism for the anticancer activity of statins in general, it is well known that high levels of mevalonate production were documented in various types of cancers. Thus, blocking the mevalonate pathway by inhibiting HMG-CoA reductase by statins, would further reduce levels of mevalonate and its downstream products (isoprenoids intermediates). Depletion of these intermediates inhibits lipid attachment sites for activated Ras, Rac, and Rho family members. These proteins have a great role in cancer formation and progression [41,42]. The enhanced in vitro cytotoxicity of the optimized SMV-SPNs against various cancer cell lines suggest that the developed formulation could possibly enhance these molecular changes significantly. To confirm this hypothesis, studying the molecular changes will be considered in future work focusing on the mechanism, including the enzyme and the involved signaling molecules.

## 4. Conclusions

The CMPV design has been successfully applied for the optimization of SMV spanlastics. The measured responses of the optimized formulation were 128.50 nm for the vesicle size, 0.329 for the PDI, and −29.11 mV for the ZP. The measured responses coincide well with the predicted ones, confirming the validity of the numerical optimization adopted in this study. The investigation of the in vitro cytotoxicity of optimized SMV spanlastics in comparison to the raw drug proved the ability of the developed formulation to enhance the anticancer activity of the drug against MCF-7, HCT-116, and HepG2 cancer cells. These results support the therapeutic potential of the SMV-SPNs against cancer, and thereby pave the way for future mechanistic studies.

## Figures and Tables

**Table 1:** MCs and PV with their ranges and response variables with their desirable constraints used in the CMPV design for the development of SMV-SPNs.

Mixture Components	Lower Level	Upper Level
X_1_: Span 60 parts	1	9
X_2_: Tween 80 parts	1	9
**Process Variable**		
Z_1_: Sonication time (min)	0	10
**Responses**	**Desirability Constraints**	
Y_1_: Particle size (PS, nm)	Minimize	
Y_2_: Polydispersity index (PDI)	Minimize	
Y_3_: Zeta potential; absolute value (ZP, mV)	Maximize	

**Abbreviations:** MC, mixture component; PV, process variable; CMPV, combined mixture process variable; SMV, simvastatin; SPNs; spanlastics.

**Table 2:** Composition and observed responses of experimental runs of SMV-SPNs prepared according to the combined mixture–process variable D-optimal design.

Run No.	Mixture Components		Process Variable	Responses ± SD		
	X_1_	X_2_	Z_1_	Y_1_	Y_2_	Y_3_
1	9	1	10	362.61 ± 15.81	0.330 ± 0.011	−30.81 ± 2.91
2	1	9	10	146.66 ± 4.99	0.290 ± 0.009	−23.80 ± 2.11
3	5	5	0	232.90 ± 10.91	0.350 ± 0.013	−28.92 ± 2.19
4	1	9	5	391.51 ± 13.72	0.312 ± 0.008	−20.60 ± 1.78
5	3	7	2.5	104.90 ± 3.11	0.216 ± 0.018	−23.70 ± 1.95
6	3	7	7.5	163.30 ± 5.89	0.390 ± 0.019	−25.70 ± 2.31
7	9	1	0	831.91 ± 26.45	0.612 ± 0.054	−29.73 ± 2.61
8	1	9	0	647.03 ± 27.98	0.447 ± 0.031	−19.40 ± 1.34
9	7	3	7.5	295.80 ± 12.34	0.220 ± 0.014	−29.80 ± 2.14
10	9	1	0	891.80 ± 36.89	0.620 ± 0.057	−31.51 ± 2.89
11	7	3	2.5	415.41 ± 15.71	0.406 ± 0.031	−28.61 ± 2.22
12	9	1	10	489.80 ± 26.56	0.240 ± 0.019	−31.70 ± 2.49
13	1	9	10	192.82 ± 11.61	0.292 ± 0.018	−23.80 ± 1.98
14	3	7	0	475.21 ± 19.87	0.316 ± 0.027	−26.10 ± 2.51
15	9	1	5	323.20 ± 13.12	0.472 ± 0.038	−28.50 ± 2.52
16	5	5	10	83.89 ± 5.31	0.341 ± 0.019	−26.9 ± 2.39
17	5	5	5	74.45 ± 3.16	0.331 ± 0.032	−28.10 ± 2.16

**Abbreviations:** SMV, simvastatin; SPNs, spanlastics; X_1_, Span parts; X_2_, Tween parts (Total parts 10); Z_1_, sonication time (min); Y_1_: particle size (nm); Y_2_, Polydispersity index; Y_3_, zeta potential (mV). Data are presented as mean of triplicate measurements of each trial ± SD.

**Table 3:** Fit statistical summary of the quadratic × linear model for SMV-SPNs responses.

Response	Model *p*-Value	Lack of Fit *p*-Value	R^2^	Adjusted R^2^	Predicted R^2^	PRESS	Adequate Precision
Particle size (PS, nm)	0.0006	0.5744	0.8217	0.7407	0.6270	2.858 × 10^5^	9.7314
Polydispersity index (PDI)	0.0005	0.1893	0.8389	0.7657	0.7080	0.085	10.3098
Zeta potential (ZP, mV)	<0.0001	0.2968	0.9383	0.9103	0.8751	26.730	17.7786

**Abbreviations:** SMV, simvastatin; SPNs, spanlastics; R^2^, multiple correlation coefficient; PRESS, predicted residual error sum of squares.

**Table 4:** Calculated IC_50_ values (μM) of SMV and SMV-SPNs in human breast, colon, and hepatic cancer cell lines.

	MCF-7	HCT-116	HepG2
**SMV**	4.850 ± 0.16	3.650 ± 0.19	1.134 ± 0.24
**SMV-SPNs**	0.8938 ± 0.27 *	0.3923 ± 0.25 *	0.0603 ± 0.15 *

**Abbreviations:** SMV, simvastatin; SPNs; spanlastics, * significantly different from SMV at *p* < 0.05.

## Data Availability

Data are contained in the article.

## References

[1] Patra H.K., Turner A.P.F. (2014). The potential legacy of cancer nanotechnology: Cellular selection. Trends Biotechnol..

[2] Mintz K.J., Leblanc R.M. (2021). The use of nanotechnology to combat liver cancer: Progress and perspectives. Biochim. Biophys. Acta Rev. Cancer.

[3] Jabir N.R., Tabrez S., Shakil S., Damanhouri G.A., Kamal M.A. (2012). Nanotechnology-based approaches in anticancer research. Int. J. Nanomed..

[4] Zhao C.Y., Cheng R., Yang Z., Tian Z.M. (2018). Nanotechnology for cancer therapy based on chemotherapy. Molecules.

[5] Sutradhar K.B., Amin M.L. (2014). Nanotechnology in Cancer Drug Delivery and Selective Targeting. ISRN Nanotechnol..

[6] Shakeel F., Alshehri S., Ibrahim M.A., Altamimi M., Haq N., Elzayat E.M., Shazly G.A. (2021). Solubilization and thermodynamic properties of simvastatin in various micellar solutions of different non-ionic surfactants: Computational modeling and solubilization capacity. PLoS ONE.

[7] El-Say K.M., Ahmed T.A., Badr-Eldin S.M., Fahmy U., Aldawsari H., Ahmed O.A.A. (2015). Enhanced permeation parameters of optimized nanostructured simvastatin transdermal films: Ex Vivo and In Vivo evaluation. Pharm. Dev. Technol..

[8] Akbarzadeh I., Saremi Poor A., Yaghmaei S., Norouzian D., Noorbazargan H., Saffar S., Ahangari Cohan R., Bakhshandeh H. (2020). Niosomal delivery of simvastatin to MDA-MB-231 cancer cells. Drug Dev. Ind. Pharm..

[9] Safwat S., Ishak R.A., Hathout R.M., Mortada N.D. (2017). Statins anticancer targeted delivery systems: Re-purposing an old molecule. J. Pharm. Pharmacol..

[10] Ali H., Shirode A.B., Sylvester P.W., Nazzal S. (2010). Preparation, characterization, and anticancer effects of simvastatin-tocotrienol lipid nanoparticles. Int. J. Pharm..

[11] Xin Y., Yin M., Zhao L., Meng F., Luo L. (2017). Recent progress on nanoparticle-based drug delivery systems for cancer therapy. Cancer Biol. Med..

[12] Tran P., Lee S.E., Kim D.H., Pyo Y.C., Park J.S. (2020). Recent advances of nanotechnology for the delivery of anticancer drugs for breast cancer treatment. J. Pharm. Investig..

[13] Piktel E., Niemirowicz K., Watek M., Wollny T., Deptuła P., Bucki R. (2016). Recent insights in nanotechnology-based drugs and formulations designed for effective anti-cancer therapy. J. Nanobiotechnol..

[14] Sapti M. (2019). Kemampuan Koneksi Matematis (Tinjauan Terhadap Pendekatan Pembelajaran Savi). Limit-Pendidik. Matemat..

[15] Habib B.A., AbouGhaly M.H.H. (2016). Combined mixture-process variable approach: A suitable statistical tool for nanovesicular systems optimization. Expert Opin. Drug Deliv..

[16] Darekar T., Aithal K.S., Shirodkar R., Kumar L., Attari Z., Lewis S. (2016). Characterization and in vivo evaluation of lacidipine inclusion complexes with β-cyclodextrin and its derivatives. J. Incl. Phenom. Macrocycl. Chem..

[17] Kakkar S., Kaur I.P. (2011). Spanlastics-A novel nanovesicular carrier system for ocular delivery. Int. J. Pharm..

[18] Badr-Eldin S.M., Aldawsari H.M., Ahmed O.A.A., Alhakamy N.A., Neamatallah T., Okbazghi S.Z., Fahmy U.A. (2021). Optimized semisolid self-nanoemulsifying system based on glyceryl behenate: A potential nanoplatform for enhancing antitumor activity of raloxifene hydrochloride in MCF-7 human breast cancer cells. Int. J. Pharm..

[19] Singh B., Bhatowa R., Tripathi C., Kapil R. (2011). Developing micro-/nanoparticulate drug delivery systems using “design of experiments”. Int. J. Pharm. Investig..

[20] Ahmed O.A.A., El-Say K.M., Aljaeid B.M., Badr-Eldin S.M., Ahmed T.A. (2018). Optimized vinpocetine-loaded vitamin E D-α-tocopherol polyethylene glycol 1000 succinate-alpha lipoic acid micelles as a potential transdermal drug delivery system: In vitro and ex vivo studies. Int. J. Nanomed..

[21] Fahmy U.A., Badr-Eldin S.M., Ahmed O.A.A., Aldawsari H.M., Tima S., Asfour H.Z., Al-Rabia M.W., Negm A.A., Sultan M.H., Madkhali O.A.A. (2020). Intranasal niosomal in situ gel as a promising approach for enhancing flibanserin bioavailability and brain delivery: In vitro optimization and ex vivo/in vivo evaluation. Pharmaceutics.

[22] Piepel G., Pasquini B., Cooley S., Heredia-Langner A., Orlandini S., Furlanetto S. (2012). Mixture-process variable approach to optimize a microemulsion electrokinetic chromatography method for the quality control of a nutraceutical based on coenzyme Q10. Talanta.

[23] Anderson M.J., Whitcomb P.J. (2000). Designing Experiments that Combine Mixture Components with Process Factors: Apply Powerful Statistical Tools to Optimize Your Formula while Simultaneously Finding the Peak Process Parameters. Chem. Eng. Prog..

[24] Sharma S., Shukla P., Misra A., Mishra P.R. (2014). Interfacial and colloidal properties of emulsified systems: Pharmaceutical and biological perspective. Colloid and Interface Science in Pharmaceutical Research and Development.

[25] Yingchoncharoen P., Kalinowski D.S., Richardson D.R. (2016). Lipid-based drug delivery systems in cancer therapy: What is available and what is yet to come. Pharmacol. Rev..

[26] Zhang Y.-R., Lin R., Li H.-J., He W., Du J.-Z., Wang J. (2019). Strategies to improve tumor penetration of nanomedicines through nanoparticle design. Wiley Interdiscip. Rev. Nanomed. Nanobiotechnol..

[27] Danaei M., Dehghankhold M., Ataei S., Hasanzadeh Davarani F., Javanmard R., Dokhani A., Khorasani S., Mozafari M.R. (2018). Impact of particle size and polydispersity index on the clinical applications of lipidic nanocarrier systems. Pharmaceutics.

[28] Elsherif N.I., Shamma R.N., Abdelbary G. (2017). Terbinafine Hydrochloride Trans-ungual Delivery via Nanovesicular Systems: In Vitro Characterization and Ex Vivo Evaluation. AAPS PharmSciTech.

[29] Abdelrahman F.E., Elsayed I., Gad M.K., Elshafeey A.H., Mohamed M.I. (2017). Response surface optimization, Ex vivo and In vivo investigation of nasal spanlastics for bioavailability enhancement and brain targeting of risperidone. Int. J. Pharm..

[30] Ghaderi S., Ghanbarzadeh S., Mohammadhassani Z., Hamishehkar H. (2014). Formulation of gammaoryzanol-loaded nanoparticles for potential application in fortifying food products. Adv. Pharm. Bull..

[31] Lasoń E., Sikora E., Ogonowski J. (2013). Influence of process parameters on properties of nanostructured lipid carriers (NLC) formulation. Acta Biochim. Pol..

[32] Fahmy U.A., Ahmed O.A.A., Badr-Eldin S.M., Aldawsari H.M., Okbazghi S.Z., Awan Z.A., Bakhrebah M.A., Alomary M.N., Abdulaal W.H., Medina C. (2020). Optimized nanostructured lipid carriers integrated into in situ nasal gel for enhancing brain delivery of flibanserin. Int. J. Nanomed..

[33] El-Helw A.R.M., Fahmy U.A. (2015). Improvement of fluvastatin bioavailability by loading on nanostructured lipid carriers. Int. J. Nanomed..

[34] Wang N., Hsu C., Zhu L., Tseng S., Hsu J.P. (2013). Influence of metal oxide nanoparticles concentration on their zeta potential. J. Colloid Interface Sci..

[35] El-Nabarawy N.A., Teaima M.H., Helal D.A. (2019). Assessment of spanlastic vesicles of zolmitriptan for treating migraine in rats. Drug Des. Dev. Ther..

[36] Van Der Weide K., Dejonge-Peeters S.D.P.W.M., Kuipers F., DeVries E.G.E., Vellenga E. (2009). Combining simvastatin with the farnesyltransferase inhibitor tipifarnib results in an enhanced cytotoxic effect in a subset of primary CD34+ acute myeloid leukemia samples. Clin. Cancer Res..

[37] Li H.Y., Appelbaum F.R., Willman C.L., Zager R.A., Banker D.E. (2003). Cholesterol-modulating agents kill acute myeloid leukemia cells and sensitize them to therapeutics by blocking adaptive cholesterol responses. Blood.

[38] Elhabak M., Ibrahim S., Abouelatta S.M. (2021). Topical delivery of l-ascorbic acid spanlastics for stability enhancement and treatment of UVB induced damaged skin. Drug Deliv..

[39] Badria F., Fayed H.A., Ibraheem A.K., Mazyed E.A. (2020). Formulation of sodium valproate nanospanlastics as a promising approach for drug repurposing in the treatment of androgenic alopecia. Pharmaceutics.

[40] Alaaeldin E., Mostafa M., Mansour H.F., Soliman G.M. (2021). Spanlastics as an efficient delivery system for the enhancement of thymoquinone anticancer efficacy: Fabrication and cytotoxic studies against breast cancer cell lines. J. Drug Deliv. Sci. Technol..

[41] Chan K.K.W., Oza A.M., Siu L.L. (2003). The Statins as Anticancer Agents. Am. Assoc. Cancer Res..

[42] Tamanoi F., Azizian M., Ashrafi M., Bathaie S. (2017). Mevalonate Pathway and Human Cancers. Curr. Mol. Pharmacol..

